# Dr. Fischer on Belladonna in Strangulated Hernia

**Published:** 1842-10-01

**Authors:** F. Fischer

**Affiliations:** Tambach


### ANALECTA.

*Injections of Belladonna in Strangulated Hernia.* By Dr. F. Fischer, of Tambach.—In September, 1838, a woman, aged 68, experienced a swelling in the right groin, during the occurrence of a severe cough; this was followed by pain and tenderness in the part, vomiting, sleeplessness, and pain in the abdomen. Examination shewed a strangulated hernia, which could not be reduced. The author directed injections of belladonna (one scruple of the leaves in each) to be administered. After three clysters the taxis was again employed, and the hernia returned without difficulty.

A man, aged forty years, had from his youth suffered under an inguinal hernia of the right side, which became incarcerated whilst he was loading a waggon with wood. After taxis, bleeding, cold applications, and other means had been tried without success, three injections of belladonna were given. The effect of these clysters shewed themselves after a while by symptoms of narcotism, as restlessness, delirium, dilated pupil, &c., which subsided under the employment of cold applications to the head. Reduction was then again attempted, and the hernia returned with very little trouble.—*Lond. Med. Gaz., from Schmidt's Jahrbücher.*

